# Nitrate transport velocity data in the global unsaturated zones

**DOI:** 10.1038/s41597-022-01621-x

**Published:** 2022-10-11

**Authors:** Congyu Yang, Lei Wang, Shengbo Chen, Yuanyin Li, Shuang Huang, Qinghong Zeng, Yanbing Chen

**Affiliations:** 1 College of Geo-exploration Science and Technology, Jilin University, Changchun, China; 2 British Geological Survey, Keyworth, Nottingham, NG12 5GG United Kingdom; 3 Department of Geography, Durham University, Durham, DH1 3LE United Kingdom; 4 MCC Smart City (Wuhan) Engineering Technology CO., Ltd, Wuhan, China

**Keywords:** Hydrology, Environmental impact

## Abstract

Nitrate pollution in groundwater, which is an international problem, threatens human health and the environment. It could take decades for nitrate to transport in the groundwater system. When understanding the impacts of this nitrate legacy on water quality, the nitrate transport velocity (*v*_*N*_) in the unsaturated zone (USZ) is of great significance. Although some local USZ *v*_*N*_ data measured or simulated are available, there has been no such a dataset at the global scale. Here, we present a **G**lobal-scale unsaturated zone **N**itrate transport **V**elocity dataset (GNV) generated from a Nitrate Time Bomb (NTB) model using global permeability and porosity and global average annual groundwater recharge data. To evaluate GNV, a baseline dataset of USZ *v*_*N*_ was created using locally measured data and global lithological data. The results show that 94.50% of GNV match the baseline USZ *v*_*N*_ dataset. This dataset will largely contribute to research advancement in the nitrate legacy in the groundwater system, provide evidence for managing nitrate water pollution, and promote international and interdisciplinary collaborations.

## Abstract (summary)

**Table:** 

Measurement(s)	Nitrate transport velocity in the unsaturated zone
Technology Type(s)	Numericial modelling including calibration using the measued/ data from literature
Factor Type(s)	Nitrate transport speed in the unsaturated zones
Sample Characteristic - Organism	None
Sample Characteristic - Environment	Groundwater pollution
Sample Characteristic - Location	Global unsaturated zones

## Background & Summary

Only 3% of the total water on the Earth is considered fresh water and approximately 30% of that is accessible as groundwater, which is vital for human development, ecosystem, the energy industry and other water-dependent activities^1^. Since the 1950s, it has been realised that nitrate (NO_3_^−^N), which is the most common groundwater pollutant worldwide^2–4^, adversely affects human health^5,6^. Studies have shown a positive correlation between nitrate concentration in drinking water and the colorectal cancer morbidity when the drinking water quality is far below the drinking water standard (50 mg/L of nitrate as NO_3_^−^ in the European Union^7^, or 10 mg/L of nitrogen as the maximum contaminant level regulated by the United States Environmental Protection Agency) set by policies^8,9^. Nitrate has also been considered to be an environmental endocrine disruptor, as it has been shown to affect vertebrate reproduction and developmental processes in fishes^10,11^. Nitrate entering wetlands, rivers or lakes can lead to eutrophication, which may lead to algae overgrowth and fish loss^12–14^. Moreover, nitrate has an indirect impact on the economy. Studies from the early 1990s showed that in response to groundwater pollution, many people took avoidance actions that can result in significant economic losses^15^. For example, in Wisconsin, USA, the direct medical cost for all adverse health consequences attributable to nitrates is estimated at between $23 million and $80 million per year^16^.

The main sources of nitrate in groundwater that cause these hazards include irrigated and rainfed agriculture and intensive animal farming^17^. Other sources, such as septic tanks and landfills, may leach nitrate locally^18^. In some urbanized areas, underground sewer leakage is also a source of nitrate in groundwater^19^. Nitrate pollution in shallow aquifers is mainly caused by fertilisation and the subsequent nitrate leaching^20,21^, which is the process of nitrate migration from the upper to the lower soil with soil water. Nitrate leaves the bottom of the soil into the unsaturated zone (USZ) and finally enters the groundwater. The USZ is located below the soil and above the groundwater, which is not only an important space connecting the surface and groundwater, but also a necessary way for all kinds of pollutants to enter the groundwater. After nitrate enters the USZ from the bottom of the soils, the transformation of nitrate in the USZ mainly includes three processes, namely, adsorption^22^, nitrification and denitrification^23^. Recent literature has indicated an increasing global concern about the effects of nitrate leaching on the environment, particularly agro-ecosystems^24^, especially the nitrate legacy in the USZ, i.e., the nitrate time lag between the bottom of the soil layer and arrival at the water table^25^. Some studies have termed this issue a ‘nitrate time bomb’^26^ and indicate that countries should consider it when assessing groundwater nitrate pollution and developing pollution control policies^27^.

To understand the nitrate legacy in the groundwater system, it is necessary to understand the nitrate transport velocity (*v*_*N*_) in the USZs and hence the nitrate lag time in the USZs. In previous studies, *v*_*N*_ was regarded as one of the main factors affecting the nitrate concentration and distribution in the USZs of the study areas^28^, and nitrate was also regarded as an environmental tracer to understand the transfer processes in the USZs^29^. However, the *v*_*N*_ in the USZs involved in these researches is limited to specific local research areas. In terms of global-scale research, there are few studies^28–37^ on *v*_*N*_ estimation, and most of the research is concentrated on European aquifers^35–37^, especially British aquifers^38^. Although *v*_*N*_ maps for the UK^38^, the Loess Plateau of China^39^ have been generated, there is no spatial map representing the *v*_*N*_ distribution for the whole world. Since the USZ *v*_*N*_ is determined by many factors, such as rock types, permeability, porosity, and amount of groundwater recharge, it is highly regional or lithological specific^40^ thus making it difficult to generate a reliable global dataset of *v*_*N*_ the USZs.

Based on a Nitrate Time Bomb (NTB) model^38^, we developed a global dataset of nitrate transport velocity in the USZs (GNV) and validated it using nitrate velocity data locally observed or derived from the literature review. This first known and open-source global-scale USZ *v*_*N*_ dataset GNV can help scientists from other disciplines to better understand the nitrate legacy in the groundwater system at a large scale, thus contributing to developing new methods to provide sound evidence for nitrate water pollution management.

## Methods

The development of the GNV consists of three steps: (1) Constructing an NTB model by preparing and inputting global datasets of rock permeability, rock porosity, and annual groundwater recharge. (2) Calibrating the NTB model based on a 22-zone baseline dataset of USZ *v*_*N*_ created using locally measured *v*_*N*_ data and global lithological data. (3) Validating the GNV dataset derived using the baseline USZ *v*_*N*_ dataset.

### The Nitrate Time Bomb (NTB) model

The NTB model has been used to simulate the nitrate transport in the groundwater system at the national and global scales^41^, based on the information on nitrate leaching from the bottom of the soils, the thickness of the USZs, and the rock hydrogeological characteristics. The NTB model was used in this study to derive the GNV dataset. The NTB model was originally developed in the UK, where the transport velocity in the USZs was calculated as the ratio of average groundwater recharge to porosity^42^:

$${V}_{USZ,i}=\frac{{R}_{ec,i}}{{P}_{rock,i}\cdot R\cdot 1000}$$

where, *V*_*USZ,i*_ (m/year) is the USZ *v*_*N*_ at the cell *i*; *R*_*ec, i*_ (mm/year) is the groundwater recharge in the cell *i*; *P*_*rock,i*_ is the porosity of the rock at the location of *i*; and *R* is the retardation factor reflecting the influences of other factors, such as permeability, pore size, diffusion, dispersion and adsorption on the USZ *v*_*N*_.

### Global porosity data for constructing the NTB model

**Fig. 1:**
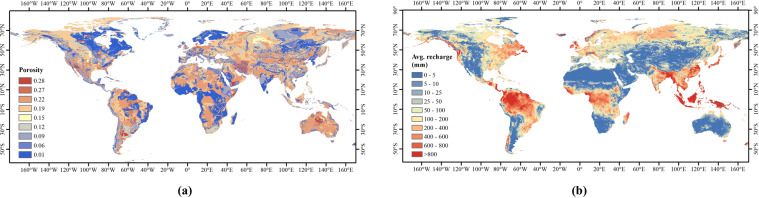
Input datasets for the NTB model. (**a**) Global porosity database. Different porosity values correspond to different lithologies, including unconsolidated sediments (0.22), coarse-grain unconsolidated sediments (0.28), fine-grain unconsolidated sediments (0.15), siliciclastic sedimentary (0.19), coarse-grain siliciclastic sedimentary (0.27), fine-grain siliciclastic sedimentary (0.12), carbonate (0.06), crystalline (0.01), and volcanic (0.09). The database is available at 10.5683/SP2/DLGXYO^66^. (**b**) Global groundwater average recharge from 1958 to 2015. The original dataset is available at https://opendap.4tu.nl/thredds/catalog/data2/pcrglobwb/catalog.html.

The global porosity data used in this study were derived from the GLHYMPS (Fig. 1a), which is global near-surface hydrogeology data of permeability and porosity produced by synthesising and modifying existing global databases^43,44^. The nine classes of porosity data, which have an average polygon size of 107 km^2^ (including Antarctica), are corresponding to nine hydrogeological categories, i.e. unconsolidated sediments, coarse-grain unconsolidated sediments, fine-grain unconsolidated sediments, siliciclastic sedimentary, coarse-grain siliciclastic sedimentary, fine-grain siliciclastic sedimentary, carbonate, crystalline, and volcanic.

### Global annual groundwater recharge data for constructing the NTB model

The global annual groundwater recharge data used in this study were derived from a global hydrological and water resource model called PCR-GLOBWB^45,46^, which has spatial resolutions of 0.5° × 0.5° and 5′ × 5′ and is available at https://github.com/UU-Hydro/PCR-GLOBWB_model^47,48^. Similar to other large-scale hydrologic models, PCR-GLOBWB is essentially a “leaky bucket” model applied on a cell‐by‐cell basis^49^ by considering rainfall, evaporation, canopy interception, snow accumulation and snowmelt. The monthly groundwater recharge derived from PCR-GLOBWB was used to calculate the annual average recharge from 1958 to 2015 (Fig. 1b).

### Regionally measured or modelled USZ *v*_*N*_ data and global lithological data for generating the global-scale baseline USZ *v*_*N*_

**Fig. 2:**
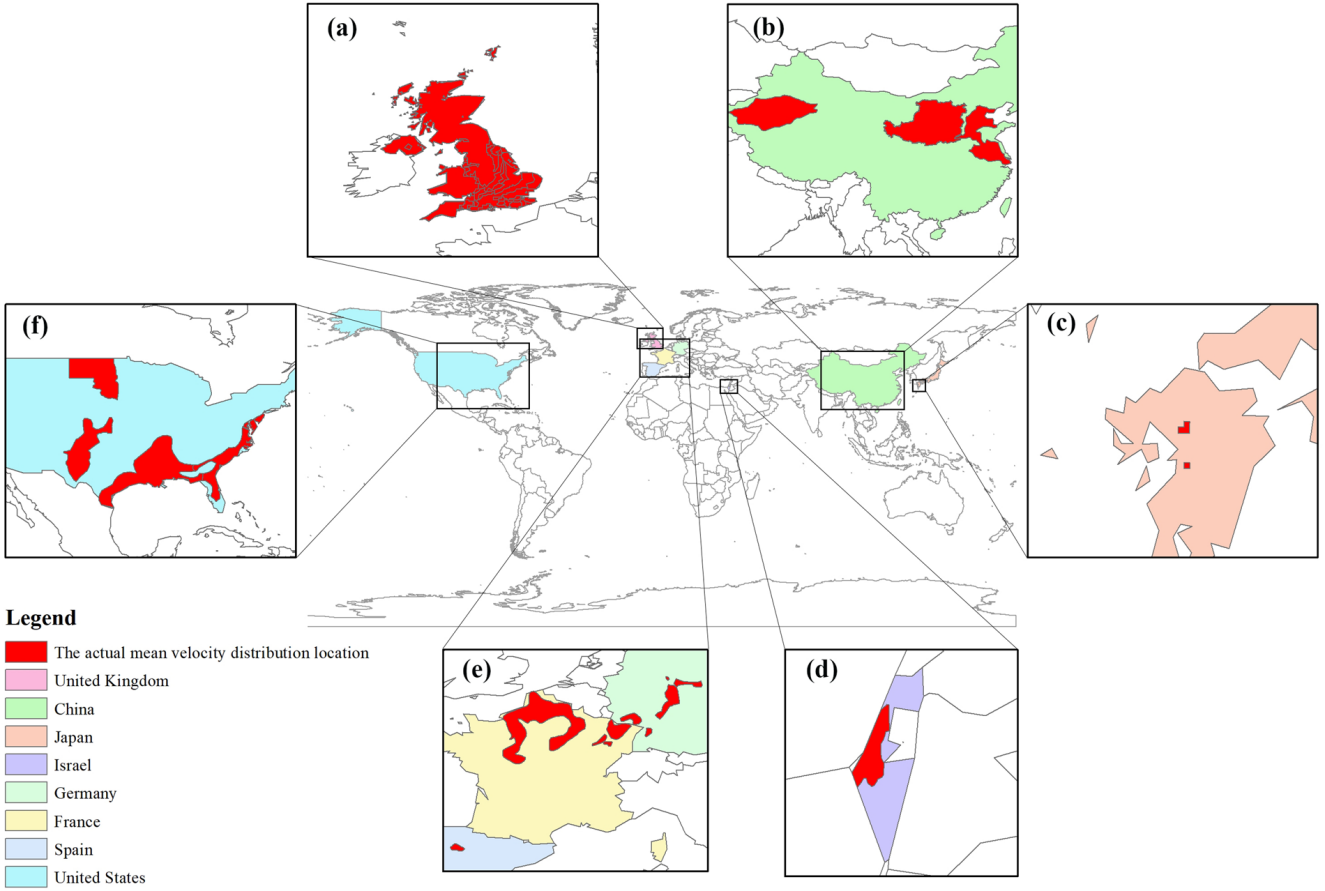
The spatial distribution of the mean USZ *v*_*N*_ data collected in this study. The red region represents the region where the mean USZ *v*_*N*_ exists (the mean USZ *v*_*N*_ maybe one or more). (**a**) The UK’s multiple mean USZ *v*_*N*_ values cover almost the whole of the UK; (**b**) The Tarim Basin, Loess Plateau and North China Plain in China have the same average USZ *v*_*N*_; (**c**) The mean USZ *v*_*N*_ of the metamorphic rocks in the Kumamoto region of Japan; (**d**) The average USZ *v*_*N*_ of the Loess in Israel; (**e**) The mean USZ *v*_*N*_ values of the Chalk and Triassic sandstone in Western Europe; and (**f**) The average USZ *v*_*N*_ of the US Loess.

To generate a global baseline dataset of USZ *v*_*N*_, the measured or modelled (but verified) *v*_*N*_ data of regional USZs in different countries were collected and averaged from published literature (Supplementary Table 1)^28–38,50,51^. Figure 2 shows the distribution of the collected mean USZ *v*_*N*_ data from the United States, China, the UK, Western Europe, Japan and Israel. These data were then expanded to a global-scale baseline USZ *v*_*N*_ dataset based on the regional lithology and the global lithology data (GLiM)^52^. GLiM, which is available at the PANGEA Database (10.1594/PANGAEA.788537)^53^, represents the rock types of the Earth surface with 1,235,400 polygons. The lithological classification consists of three levels: the first level contains 16 basic lithological classes, while the other two levels contain 12 and 14 subclasses respectively describing more rock details. Only 16 basic classes of the first level of GliM were used in this paper, including: Intermediate volcanic rocks, Basic volcanic rocks, Acid plutonic rocks, Metamorphics, Unconsolidated sediments, Siliciclastic sedimentary rocks, Basic plutonic rocks, Intermediate plutonic rocks, Mixed sedimentary rocks, Water Bodies, Pyroclastics, Carbonate sedimentary rocks, Acid volcanic rocks, No Data and Evaporites. According to the GLiM, the Earth is covered by 64% sediments (a third of which are carbonates), 13% metamorphics, 7% plutonics, 6% volcanics, and 10% are covered by water or ice^52^.

### Baseline datasets of USZ *v*_*N*_ for calibrating and validating the NTB model

**Fig. 3:**
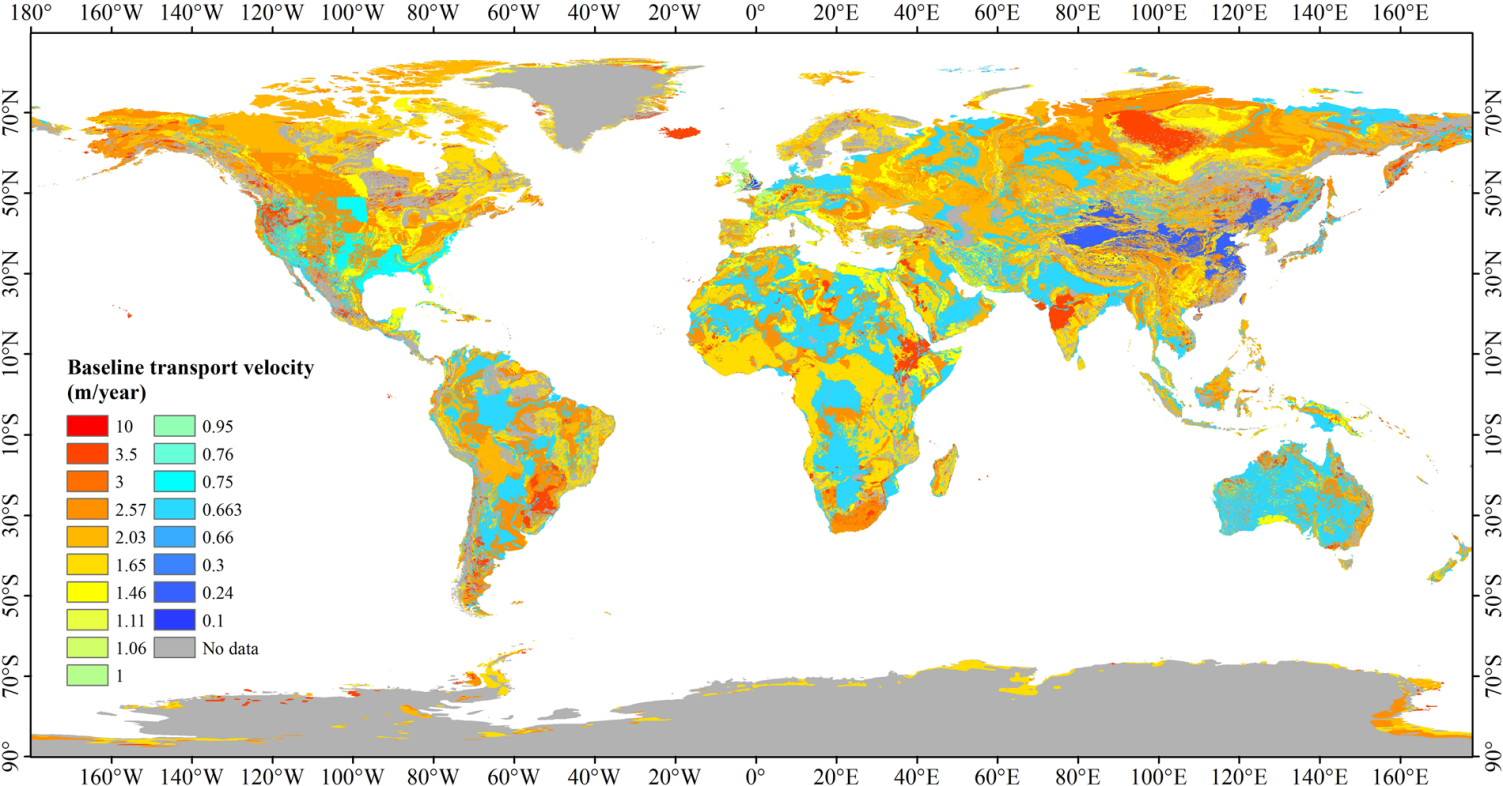
The baseline USZ *v*_*N*_ data that contain the average nitrate transport velocities measured or modelled in the regions.

**Fig. 4:**
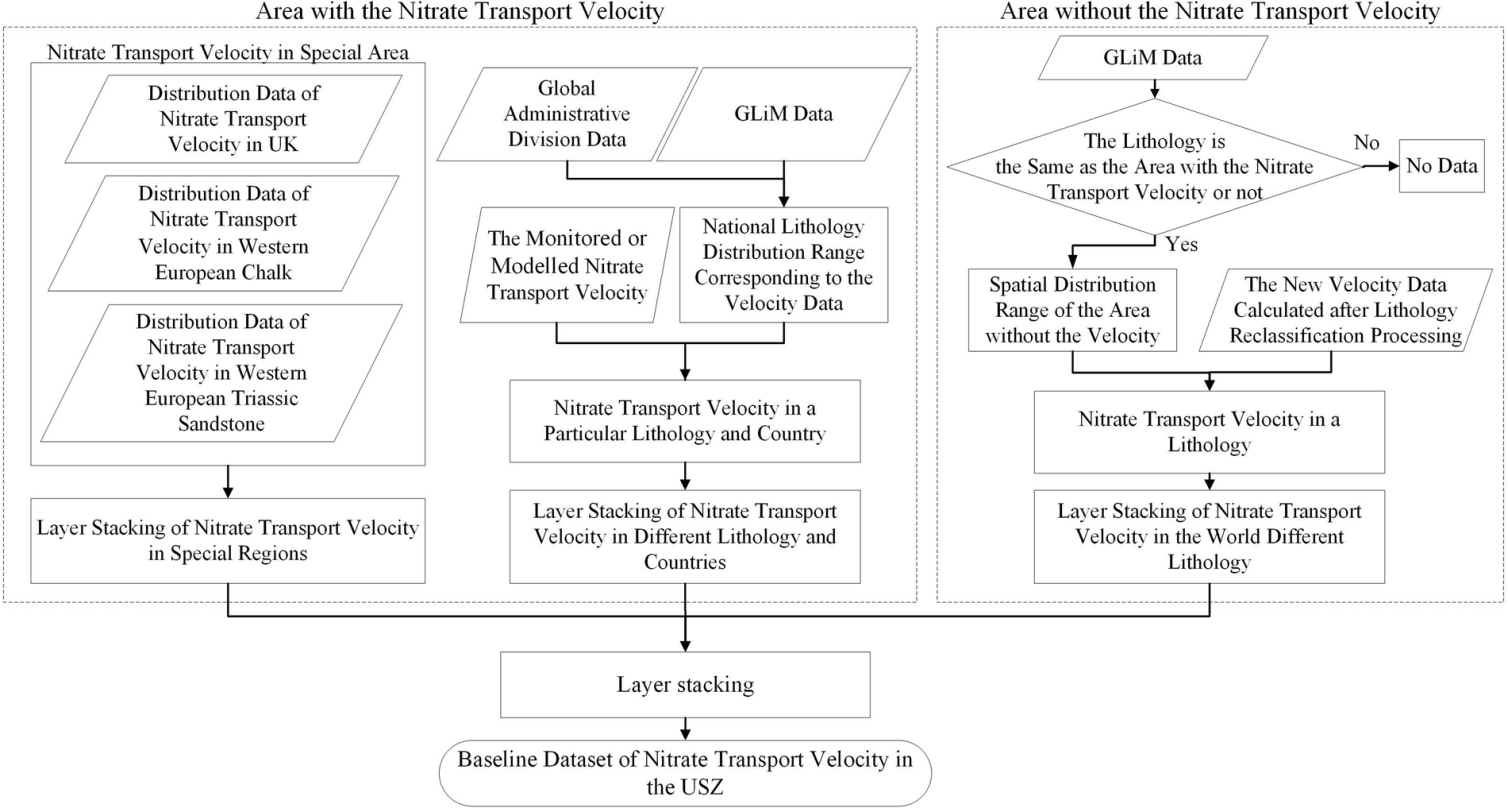
The flowchart of generating a global baseline dataset of USZ *v*_*N*_.

The first level of GLiM classification was used in this study as a base map to interpolate the regionally measured or modelled (but verified) USZ *v*_*N*_ data into a global baseline dataset of USZ *v*_*N*_ (*v*_*N_base*_) (Fig. 3), which is used as observed/known *v*_*N*_ to calibrate the NTB model. Figure 4 shows the flowchart of generating the *v*_*N_base*_. According to the principle of the NTB model, the *v*_*N*_ is constrained by USZ lithology conditions, so we assumed that the same USZ lithology had the same average USZ *v*_*N*_. The collected regional monitored or modelled USZ *v*_*N*_ data and their corresponding USZ lithologies were collated, and the world was divided into two parts according to the existence of USZ *v*_*N*_, namely, the regions with and without *v*_*N*_ data. For the regions with *v*_*N*_ data, we divided them into regions with different lithology classifications and then reclassified the lithology of these regions based on the GLiM classification (Supplementary Table 2), to calculate the mean *v*_*N*_ values of the reclassified lithology. Whilst, for the regions without *v*_*N*_ data, we derived the *v*_*N*_ values based on the lithology types that are the same as that in regions with *v*_*N*_ data. Finally, the global baseline dataset of USZ *v*_*N*_ was generated using the mean *v*_*N*_ data from all the regions.

The data processing of monitored or modelled USZ *v*_*N*_ collected was mainly divided into three parts: (A) the USZ *v*_*N*_ in the UK; (B) the USZ *v*_*N*_ in Chalk and Triassic sandstone of Western Europe; and (C) the USZ *v*_*N*_ in other regions. Their data processing are described below:

(A) Since the UK has a complete database of USZ *v*_*N*_ with a detailed description of aquifers that cover almost the whole country^38^, this UK database was used to derive the mean USZ *v*_*N*_ of other regions in the world based on aquifer types. Therefore, the UK aquifers were reclassified using the basic lithological classification standard of the global GLiM data (Supplementary Table 2). For example, according to the spatial distribution, Chalk, Carboniferous, Cornbrash and Great Oolite of Lincolnshire and other lithology in the UK belong to the Carbonate sedimentary rocks defined in the GLiM basic lithology. Lower Cretaceous Sands, Triassic Sandstones, Triassic and Permian and other lithology belong to the Siliciclastic sedimentary rocks of the GLiM basic lithology. The Pliocene: Corralline Crag and Quaternary Norwich and Red Crags belong to the Mixed sedimentary rocks of the GLiM basic lithology. When more than one UK USZ lithologies were classified into one GLiM lithological type after the reclassification, the mean *v*_*N*_ value of these USZ lithologies was calculated and applied to calculate the mean USZ *v*_*N*_ of other parts of the world.
(B) Because of special lithological classifications in Western Europe^54^ (Belgium, the former Federal Republic of Germany, Denmark, France, Ireland, Italy, Luxembourg, the Netherlands and the United Kingdom), the *v*_*N*_ values in USZs of the Chalk and Triassic sandstone in Western Europe were determined based on the lithological classification of Western Europe.
(C) Regarding other countries that have USZ *v*_*N*_ collected from literature, the World Administrative Region data^55,56^ and the lithology classification of GLiM were used to extrapolate the USZ *v*_*N*_ values at the study areas in the literature to the lithological range within the boundary of the countries where the studies were undertaken (Supplementary Table 2).

**Fig. 5:**
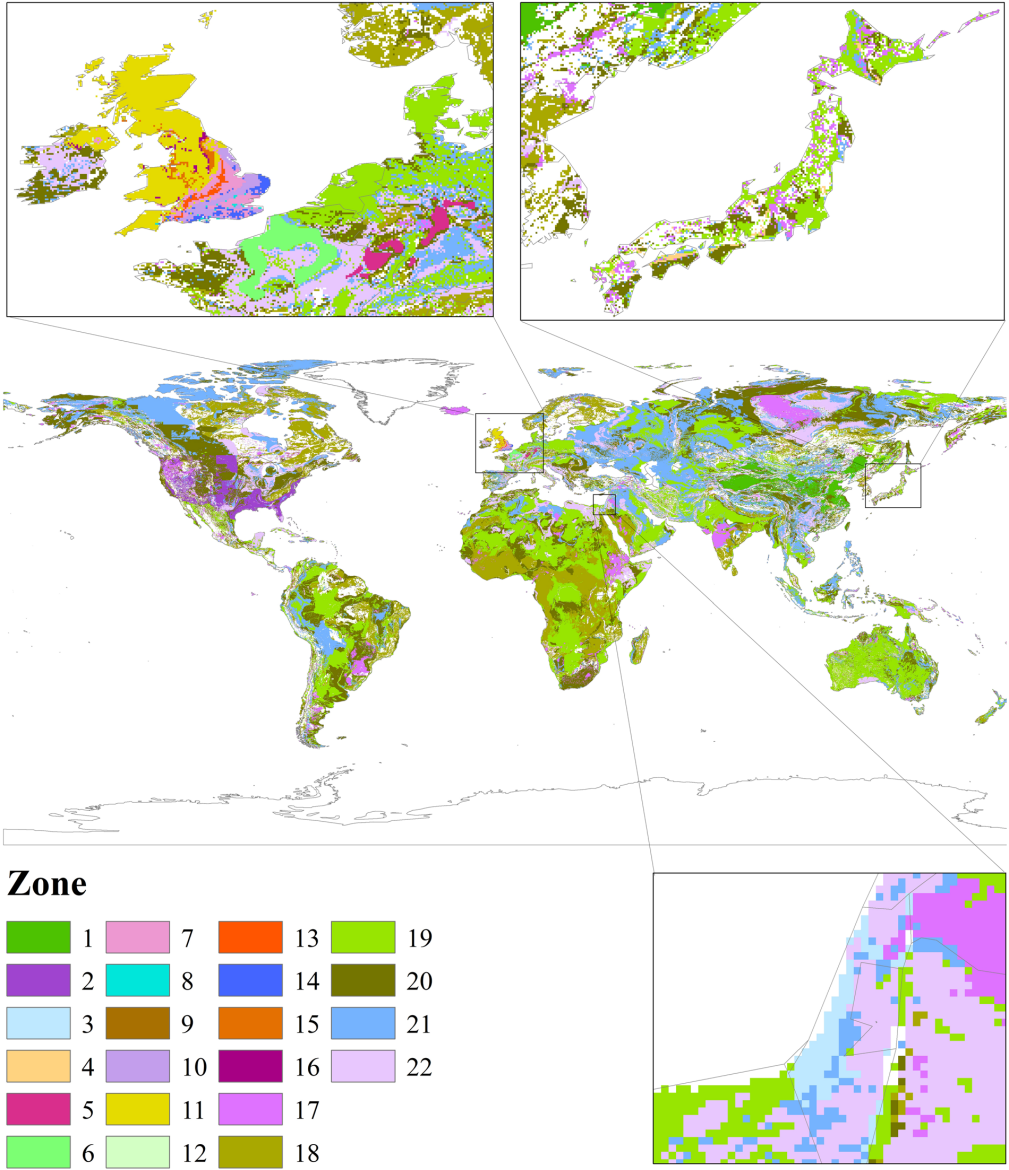
The global 22 USZ zones that exclude the areas covered by water, ice and snow, or have no *v*_*N_base*_ values.

The main factors affecting the value of the retardation factor *R* include permeability, pore size, diffusion, dispersion and adsorption, which are constrained by lithology^42^. To accurately simulate spatially distributed USZ *v*_*N*_ values, the *R* values for different lithological classifications need to be calibrated using *v*_*N_base*_. Therefore, according to the GLiM lithology classification and the *v*_*N_base*_ of different countries, the global USZs were divided into 22 zones (excluding water, ice and snow, and no *v*_*N_base*_ value zones) (Fig. 5). The zoning method is as follows: for the whole country where there is a mean USZ *v*_*N*_ and the *v*_*N_base*_ in the area is the mean USZ *v*_*N*_ (e.g., the UK), we divide the region with the same *v*_*N_base*_ into one zone. For areas where there is a mean USZ *v*_*N*_ of lithology, the *v*_*N_base*_ in the area is the mean USZ *v*_*N*_ and the lithology boundary across several countries (such as Chalk and Triassic sandstone in Western Europe), we divide the lithology into one zone according to the boundary. For areas where there is a mean USZ *v*_*N*_ of lithology, the *v*_*N_base*_ in the area is obtained by using GLiM lithology to expand the space according to the subordinate relationship between the lithology and GLiM 16 lithology, and the lithology boundary exists only in one country (such as China, the United States, Japan and Israel), we divide the GLiM lithology corresponding to this lithology in this country into one zone. For example, there is a mean USZ *v*_*N*_ in the Loess of China, and the loess region belongs to the unconsolidated sediments of GLiM. We divide the unconsolidated sediments of China into one zone. The other areas where there is no mean USZ *v*_*N*_ and the *v*_*N_base*_ is obtained by interpolation are divided according to GLiM lithology. The division of 22 zones is based on the existence of the mean USZ *v*_*N*_ data, the calculation method of *v*_*N_base*_ and lithology. Compared with GLiM 16 lithology classifications, the 22-zone zoning method distinguishes the region where *v*_*N_base*_ is obtained by using different methods according to the mean USZ *v*_*N*_ in the same lithology, to better restrict the value of retardation factor of *v*_*N_base*_ directly obtained from the existence of mean USZ *v*_*N*_ in the region, thus increasing the accuracy of the velocity simulation results. The number and the lithology of the 22 zones are shown in Supplementary Table 3. The zone map provided regional constraints for deriving spatially distributed USZ *v*_*N*_ values using the NTB model.

### Generating the global distributed USZ *v*_*N*_ data (GNV)

**Fig. 6:**
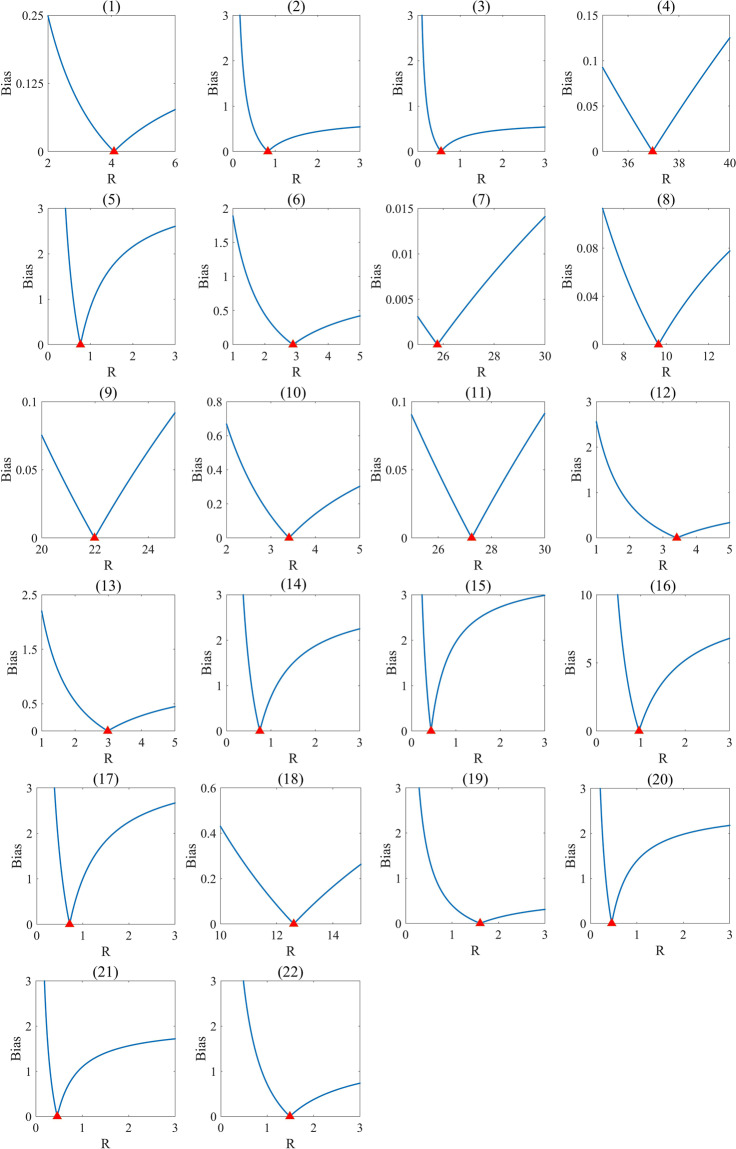
Sensitivity scatter plots for 22 zones. The bias values are the absolute values of the differences between the mean simulated results and the baseline values (*v*_*N_base*_); the blue lines consist of dots representing the bais values of 100,000 MC runs; the red triangles are the best retardation factor (*R*) values for 22 modelling zones (**1**–**22**).

**Fig. 7:**
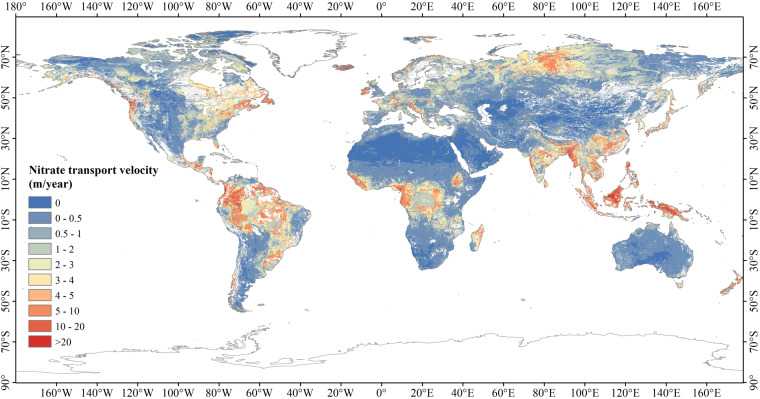
Global distributed USZ *v*_*N*_ data (GNV) generated in this study.

Although some regional monitored USZ *v*_*N*_ data can be found from published literature, the number of these data are too limited to be directly used to derive the spatially distributed USZ *v*_*N*_ values in the rest of the regions of the world. However, these collected regional monitored USZ *v*_*N*_ data have been used to derive the baseline datasets of USZ *v*_*N*_, i.e., the *v*_*N_base*_ dataset for calibrating the NTB model, which was used in this study to generate the global distributed USZ *v*_*N*_ data (GNV). To calibrate the NTB model using *v*_*N_base*_ in 22 zones (described in the section above), the different values of NTB retardation factors *R* were used and calibrated in each zone during the Monte Carlo (MC) simulation, in which, the NTB model was run 100,000 times. In each NTB run, the absolute value of the difference between the baseline datasets and the spatially distributed mean simulated values was calculated to verify the accuracy of the modelled results. The sensitivity scatter plots of the 22 zones were produced by plotting the absolute value of the difference between the baseline datasets and the spatially distributed mean simulated values of the NTB retardation factors (Fig. 6). For example, in Fig. 6(1), the number 1 corresponded to the zone1. We used the MC method to enter a random *R* value as *R*_*i*_, ran the NTB model once, and got a mean simulated velocity (*v*_*N_sim*_) of the zone1. The absolute value of the difference between the mean *v*_*N_sim*_ and the zone1 *v*_*N_base*_ was marked with a blue point in Fig. 6(1). The MC model had been run for 100,000 times and a total of 100,000 *R*_*i*_ and 100,000 scatter points had been obtained. Among these scatter points, the point with the value closest to 0 indicated that the mean *v*_*N_sim*_ is closest to the *v*_*N_base*_, and we called this mean *v*_*N_sim*_ as the best mean *v*_*N_sim*_. The *R*_*i*_ corresponding to this point was the best *R* of zone1, marked with a red triangle in Fig. 6(1). The values of *v*_*N_base*_ the simulated velocity (*v*_*N_sim*_) and retardation factor in 22 regions are shown in Supplementary Table 3. After the *R* values of 22 zones were determined, the NTB model was run again, and the GNV dataset was obtained. The GNV dataset generated using the NTB model is shown in Fig. 7.

## Data Records

The GNV dataset and its quality details are made available to the public free of charge in GeoTIFF format through an unrestricted public repository (Figshare^57^). The data is provided in a 5′ × 5′ spatial resolution with the velocity unit of m/year. The GNV dataset represents the global distribution of USZ nitrate transport velocities, which are mainly affected by rock types, rock hydrogeological characteristics, long-term groundwater recharge, etc. Data quality information, which will be discussed in the following section, is the precision estimation of nitrate transport data based on the *v*_*N_base*_ values in different zones. Upon the availability of new regionally measured or modelled USZ *v*_*N*_ data, the repository will be updated with a newer version of the nitrate transport data graph.

## Technical Validation

**Fig. 8:**
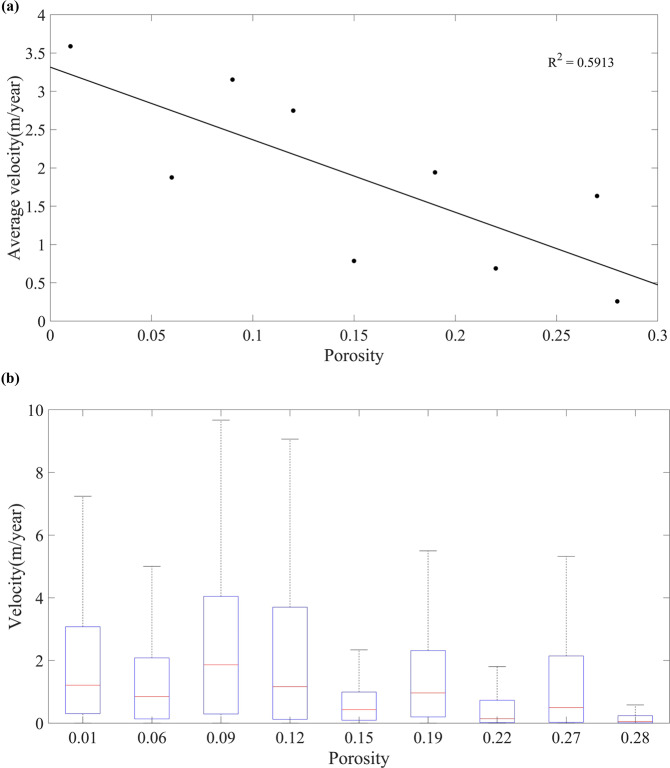
Comparison of the global rock porosity data with the *v*_*N*_. (**a**) the points in the plot represent the average *v*_*N*_ in different porosity zones and their corresponding porosity values; (**b**) the statistical distribution of *v*_*N*_ values at different porosity values.

**Fig. 9:**
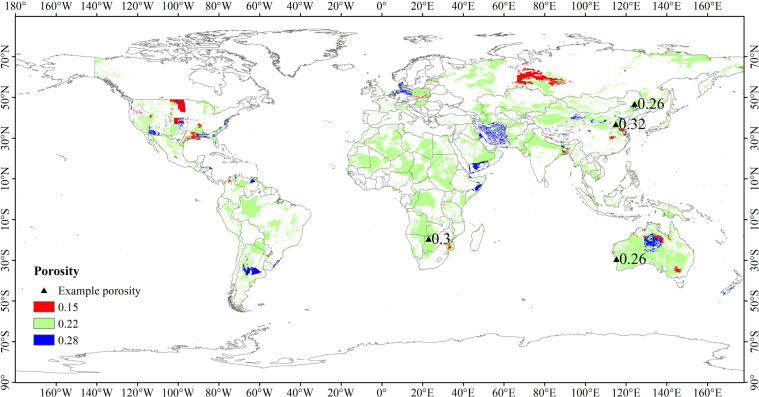
The distribution of the example porosity values. The NTB model uses porosity values of 0.15, 0.22 and 0.28; and the triangle represents porosity values from literature.

Since the global rock porosity dataset is one of the input parameters when estimating USZ *v*_*N*_ in the NTB model, the correlation analysis of USZ *v*_*N*_ distribution and porosity data are performed. In order to eliminate the influence of zero groundwater recharge on this analysis, the zero USZ *v*_*N*_ results calculated by zero average annual groundwater recharge (e.g. Sahara Desert, Arabian Desert, Iranian Desert, Turkish Desert, Taklimakan Desert, Gobi Desert, Australian Desert, Namib Desert and Karari Desert) were not considered. Figure 8a shows that the mean value of *v*_*N*_ is inversely proportional to the rock porosity on the whole; and this is consistent with the basic formula of the NTB model^42^. However, when the porosity values are 0.15, 0.22 and 0.28, the value ranges of *v*_*N*_ are smaller than that of other porosity values (Fig. 8b). To explain this phenomenon, we checked the lithology classification of GLiM and found that these three porosity values belong to the same lithology category, namely unconsolidated sediment^58^. We compared the spatial distribution of these three kinds of porosity with the spatial distribution^59^ of unconsolidated sediment subtypes in the Global unconsolidated sediment Map Database (GUM)^58^. Through statistical comparative analysis, it was found that under the condition of excluding undifferentiated sediments, the unit area of clay and silt (assuming that different particles in the mixture were mixed in the same volume) corresponding to these three kinds of porosity accounts for more than 30% of the corresponding porosity area (Table 1). This shows that there was a large amount of clay and silt in the unconsolidated sediments with porosity values of 0.15, 0.22 and 0.28. The reference values of porosity of clay and silt are 0.4~0.7 and 0.8 respectively^60^, which are much higher than the 0.15, 0.22 and 0.28 used to calculate the *v*_*N*_. In order to verify the accuracy of this conclusion, we obtained the example porosity values from literature (Fig. 9)^61–64^. Figure 9 shows that it is possible that the actual porosity values can be higher than that used in the NTB model. Based on the above analysis, the actual porosity of the unconsolidated sediments may be higher than 0.15, 0.22 and 0.28 used in the NTB model, thus leading to overestimating *v*_*N*_. However, the *v*_*N*_ calculation uncertenties, which were introduced by porosity errors, can be reduced due to the existence of the retardation factor in the NTB model. When calibrating the NTB model using the baseline *v*_*N*_, the retardation factor can be adjusted to make the *v*_*N*_ modelled closer to the real *v*_*N*_.

**Table 1:** When the porosity values are 0.15, 0.22 and 0.28, the corresponding type, particle size and unit proportion of unconsolidated sediments are presented.

Porosity	Grain size	Sediment subtype^59^	Unit proportion(%)	Clay&Silt proportion(%)
0.15	Sand+	Glacio-fluvial	7.17	41.81
		Till	24.23	
	Sand/Silt	Loess derivative	24.57	
	Sand/Clay	Fluvial-lacustrine	29.01	
	Silt	Loess	15.02	
0.22	Sand+	Alluvial terrace deposits	1.83	42.99
		Dune sands	18.22	
		Glacio-fluvial	4.93	
		Till	9.65	
	Sand/Silt	Loess derivative	7.86	
		Peat	5.36	
	Sand/Clay	Fluvial-lacustrine	19.31	
		Alluvial/Colluvial	12.23	
	Silt	Loess	10.29	
	Silt/Clay	Fluvial-eolian	2.43	
		Glacio-lacustrine	0.49	
		Salt	1.34	
	Clay	Floodplain deposits	6.06	
0.28	Sand+	Dune sands	68.66	31.34
	Silt	Loess	31.34	

In Table 1, Unit proportion and Clay&Silt proportion represent statistics for Sediment subtype units based on data presented in Fig. 1a and the GUM database. Unit proportion is the proportion of the grid unit area of unconsolidated sediments subtype classification to the grid unit area of the corresponding porosity. Clay&Silt proportion is the proportion of the total grid unit area of clay and silt to the grid unit area of the corresponding porosity.

**Fig. 10:**
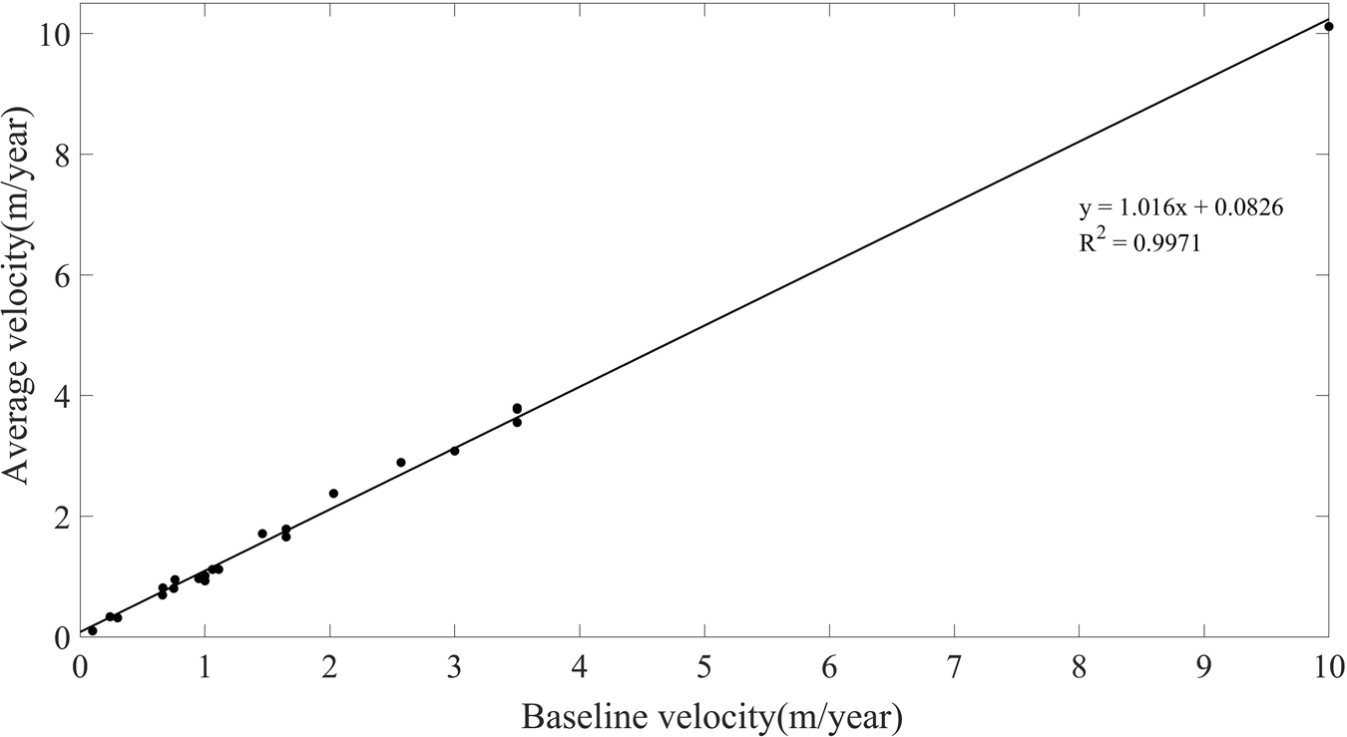
The scatterplot of correlation between the average GNV and the *v*_*N_base*_.

**Fig. 11:**
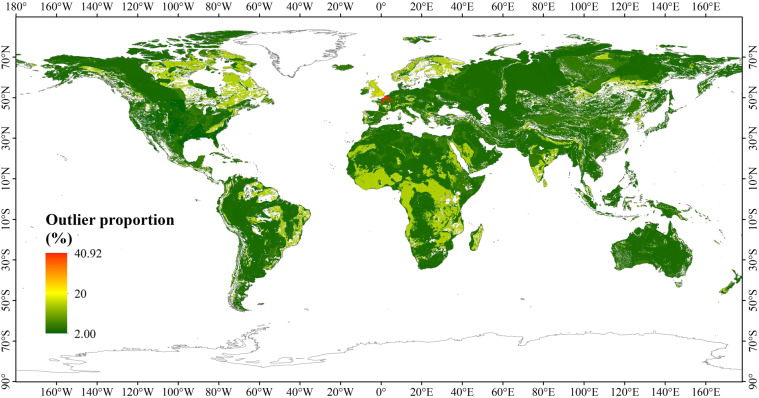
Spatial distribution of the outlier proportions (shown in Table 3).

To verify the accuracy of the GNV dataset derived in this study, we compared the simulation results with the baseline velocity in 22 zones. Firstly, the average value of simulation results in each region was compared with that of the *v*_*N_base*_. Table 2 shows that the maximum error between the average value of *v*_*N*_ and *v*_*N_base*_ is 0.4252 in zone 21, which has the main lithology of the mixed sedimentary rocks. The scatter plot of correlation between the average *v*_*N*_ and *v*_*N_base*_ shows a strong positive correlation (Fig. 10, R^2^ = 0.9956). To further evaluate the accuracy of the GNV data, the *v*_*N_base*_ ± the standard deviation of *v*_*N*_ in each zone were taken as the confidence interval, and the *v*_*N*_ values outside the confidence interval were taken as the outliers, and then the cell proportion of outliers in each region was calculated (Table 3). The results show that zone 6, which has the lithology of Chalk in Western Europe, has the largest proportion of outliers (40.92%). Besides, the outlier proportions in zone 8, 9, 10 and 13 are also relatively large, accounting for 32.14%, 25%, 24.25% and 25.93%, respectively. However, outliers in these regions only occupy a small proportion globally. Therefore, the overall percentage of outliers is 5.50%, indicating that the accuracy of GNV is 94.50%. Figure 11 shows the outlier proportion of each zone. It can be found that the outliers in Western Europe and southern Britain have relatively large proportions. This is because the total areas of these regions are comparatively small, and a single grid cell takes up a large proportion of the region, resulting in a relatively high proportion of outliers.

**Table 2:** The maximum (Max-*v*), minimum (Min-*v*), and average (Avg-*v*) values of *v*_*N*_ and the difference between the average *v*_*N*_ and the *v*_*N_base*_ in 22 zones, based on data presented in Fig. 5 and the GNV dataset presented in Fig. 7.

Zone	Max-*v* (m/year)	Min-*v* (m/year)	Avg-*v* (m/year)	difference
1	54.3173	0.0000	0.3354	0.0954
2	208.5967	0.0000	0.8044	0.0544
3	4.8257	0.0000	0.6954	0.0354
4	10.7158	0.0000	1.6582	0.0082
5	43.7150	0.0137	3.7747	0.2747
6	3.2351	0.0516	0.9301	−0.0699
7	0.5658	0.0226	0.1009	0.0009
8	0.6267	0.0972	0.3168	0.0168
9	3.0684	0.1431	0.9500	0.1900
10	2.4700	0.1655	0.9677	0.0177
11	10.0681	0.0000	1.0141	0.0141
12	3.4465	0.5573	1.1189	0.0589
13	2.4656	0.1783	1.1203	0.0103
14	12.8182	0.6826	3.0822	0.0822
15	14.6052	1.2340	3.5547	0.0547
16	68.8014	0.7753	10.1167	0.1167
17	327.5643	0.0000	3.7971	0.2971
18	33.7497	0.0000	1.7879	0.1379
19	166.7290	0.0000	0.8138	0.1508
20	512.0000	0.0000	2.8921	0.3221
21	588.2890	0.0000	2.3786	0.3486
22	168.9150	0.0000	1.7125	0.2525

**Table 3:** The standard deviation of the *v*_*N*_, confidence intervals, and outlier proportions for 22 zones.

Region	Standard deviation	Confidence interval(m/year)	Outlier proportion (%)
1	1.2045	(0.0000,1.4445)	5.23
2	3.6148	(0.0000,4.3648)	2.00
3	0.8672	(0.0000,1.5272)	8.25
4	1.5315	(0.1185,3.1815)	15.00
5	7.3985	(0.0000,10.8985)	4.82
6	0.5110	(0.4890,1.5110)	40.92
7	0.0651	(0.0349,0.1651)	17.57
8	0.1614	(0.1386,0.4614)	32.14
9	1.4143	(0.0000,2.1473)	25.00
10	0.3603	(0.5897,1.3103)	24.25
11	1.6345	(0.0000,2.6345)	16.22
12	0.5917	(0.4683,1.6517)	13.33
13	0.4810	(0.6290,1.5910)	25.93
14	2.5857	(0.4143,5.5857)	18.09
15	2.5848	(0.9152,6.0848)	11.83
16	9.1387	(0.8613,19.1387)	10.47
17	8.5980	(0.0000,12.0980)	4.98
18	2.0383	(0.0000,3.6883)	15.02
19	2.9207	(0.0000,3.5837)	3.26
20	9.2670	(0.0000, 11.8370)	2.96
21	8.0919	(0.0000, 10.1219)	3.35
22	3.6457	(0.0000, 5.1057)	5.75
Total			5.50

Standard deviation represents the standard deviation for the zones based on data presented in Fig. 5 and the GNV based on data presented in Fig. 7. Since there is no negative nitrate transport velocity, when the confidence interval endpoint appears negative, the endpoint value is replaced by 0 value.

## Usage Notes

In this paper, the global-scale USZ *v*_*N*_ dataset named GNV was generated using the NTB model based on the global porosity data and global groundwater average recharge datasets from 1958 to 2015. This GNV dataset was derived by constraining the NTB model and has been carefully analysed and verified using the measured values in various regions of the world from published literature.

Generally, the information on nitrate transport velocity in the USZs is valuable when better understanding the legacy of nitrate in the groundwater system and investigating and forecasting its impacts on nitrate in groundwater on the environment, human health, ecological quality, plant and animal growth. In detail, the GNV dataset can be used by different numerical models, such as groundwater and USZ pollution transport models and surface water models, in conjunction with other datasets. For example, the GNV dataset can be combined with the USZ thickness data to calculate the lag-time in the USZs (the time for nitrate to travel from the bottom of the soils to the water table). Similarly, this GNV dataset could be used to estimate the time when the peak value of nitrate leaching reaches the water table, thus informing policymakers to be prepared for the possible increase or decline of nitrate concentrations in groundwater in the future.

This global study can help funders, policymakers and practitioners of a country better understand the feasible time scale for expecting the benefits of nitrate mitigation measures, thus guiding setting regional priorities of groundwater nitrate management plans at the country scale. However, further localised work needs to be undertaken to get detailed information when handling local groundwater nitrate pollution issues.

This calibrated GNV dataset is available in GeoTIFF and ASC formats, thus making it easy to be imported into ESRI ArcMap and any other geospatial software.

The limitation of this study is that deriving the GNV dataset relies on global annual groundwater recharge and global porosity data, thereby possibly passing the uncertainties in these two datasets to this GNV dataset. Besides, 8.30% of the global area in GNV have no values of nitrate velocity in the USZs due to the lack of measured *v*_*N*_ data for the rock types in these areas. According to the classification of 16 basic lithological types of GLiM, the lithological classes, which have no measured USZ *v*_*N*_, includes Intermediate volcanic rocks, Acid plutonic rocks, Basic plutonic rocks, Intermediate plutonic rocks, Pyroclastics, Acid volcanic rocks and Evaporites. However, these data can be updated once the measured USZ *v*_*N*_ for the rocks in these areas become available.

## Supplementary information

Supplementary Table 1

Supplementary Table 2

Supplementary Table 3

## Data Availability

The NTB model code involved in generating GNV was developed using VC++, and the code is available in Figshare^65^. Usage methods and important parts of the code have been commented.
