# Supplementary material for: Nitrate transport velocity data in the global unsaturated zones
Source: Sci Data. 2022 Oct 11;9:613. doi: 10.1038/s41597-022-01621-x (PMC9553929; doi:10.1038/s41597-022-01621-x)
Supplement: Supplementary file 1 — Supplementary Table 1 [file 41597_2022_1621_MOESM1_ESM.docx]

| **Lithology** | **Lithological information** | **Region** | **Mean velocity**  **(m/year)** | **Reference** |
| --- | --- | --- | --- | --- |
| Loess | Homogeneous, porous, friable, pale yellow or buff, slightly coherent, typically non-stratified, and often calcareous. | The Loess Plateau, China | 0.24 | Baran et al.  Huang et al. |
|  |  | The North China Plain |  |  |
|  |  | Tarim river basin, China |  |  |
| Loess | Include 30%sand, 40%silt, 30%clay and 20%calcareous. | Moshav Omer, Israel | 0.66 | Gvirtzman & Magaritz. |
| Loess | Mainly include sandy silts and sand. | United States | 0.75 | Alberts & Spomer .  Bobier et al. |
| Mesozoic metamorphic and igneous rocks | Include sandy clay, clay sand, volcanic ash sand, volcanic ash clay, gravelly clay, non-welded tuff, and gravel. | Kumamoto region, Japan | 1.65 | Okumura et al. |
| Chalk | Porosity is estimated at 42 ± 2%. The specific yield of the matrix is between 1% and 4%, with a hydraulic conductivity estimated at 10−3 m/day. The hydraulic conductivity associated with fractures is between 10−1 and 102 m/day. | Western Europe | 1 | Chen.  Brouyère et al.  Geyh. |
| Triassic sandstone | The superficial cover is generally thin; comprising Till deposits, less than 2m thick, which occur over 60% of the bedrock. | Western Europe | 3.5 | Butcher A et al. |
| Chalk | A very fine-grained (less than 10 µm), pure (c. 98% CaCO_3_), soft, white limestone containing some marl bands and flint. | United Kingdom | 0.95 | Butcher A et al.  Wang et al.  Bogardi et al.  Allen et al. |
| White Chalk Subgroup | Chalk with flints. With discrete marl seams, nodular chalk, sponge-rich and flint seams throughout. | United Kingdom | 0.76 |  |
| Carboniferous: Limestone and Basal conglomerate | Include mudstones, siltstones and sandstones in addition to the limestones. | United Kingdom | 1 |  |
| Lower Cretaceous Sands | Include wellrounded to sub-rounded quartz grains in a micromass of quartz and clays. | United Kingdom | 3 |  |
| Cornbrash, Great Oolite and Inferior Oolite | Include soft limestone and oolitic limestones. | United Kingdom | 1 |  |
| Upper Lias: Bridport, Midford, Yeovil and Cotteswold Sands | Include yellow sand with doggers (beds of calcareous sandstone). | United Kingdom | 0.1 |  |
| Triassic Sandstones | Include limestones, dolomites, evaporites, sandstones and siltstones. | United Kingdom | 3.5 |  |
| Lower Carboniferous: Scremerston Group and Fell Saandstone of N England | Include sandstone, siltstone and mudstone. | United Kingdom | 1 |  |
| Corallian | Include fine-grained calcareous sandstones , sandy limestones, hard grits with subsidiary limestones and oolitic limestones. | United Kingdom | 1 |  |
| Millstone Grit series of Cumbria, Durham and Northumberland | Include sandstones, mudstones and siltstones. | United Kingdom | 1 |  |
| New Red Sandstone of SW England, Permian Sands of NW England | Include mudstones, reddish to yellowish sandstones, and interbedded with rare evaporite minerals such as halite and gypsum. | United Kingdom | 1.06 |  |
| Millstone Grit Series | Include sandstones, mudstones and siltstones. | United Kingdom | 1 |  |
| Upper Greensand | Consist mainly of ‘malm-stone’ formed by cemented sand; a pale coloured rock containing abundant sponge spicules and a high proportion of colloidal silica, calcareous material and some clay and mica. | United Kingdom | 3 |  |
| Magnesian Limestone | Include calcareous and dolomitic siltstones, dolomitic carbonates, mudstones and siltstones. | United Kingdom | 10 |  |
| Inferior Oolite: Lincolnshire Limestone | Limestone, typically calcilutites, and peloidal wackestones and packstones in the lower part and high energy ooidal and shell fragmental grainstones in the upper part. | United Kingdom | 1.11 |  |
| Purbeck Beds and Portland Beds | Include calcareous mudstone, limestone, clays, shales and marls with marly. | United Kingdom | 1 |  |
| Cornbrash and Great Oolite of Lincolnshire | Include limestone. | United Kingdom | 1.11 |  |
| Whin Sill | Include dolerite. | United Kingdom | 1 |  |
| Pliocene: Coralline Crag | Calcarenite, and include bioclastic calcarenites and silty sands with shell debris. | United Kingdom | 3 |  |
| Upper Coal Measures: Pennant Sandstone of South Wales | Include sandstones and mudstones. | United Kingdom | 1 |  |
| Wealden: Hastings Beds | Include sands and clays. | United Kingdom | 3 |  |
| Cornbrash, Great Oolite, Fullers' Earth and Inferior Oolite of S England | Include soft limestone and oolitic limestones. | United Kingdom | 1.11 |  |
| Oligocene: Bovey Beds | Include sands, clays and lignite. | United Kingdom | 0.3 |  |
| Quaternary Norwich and Red Crags | Include sand, gravel, clay and silt. | United Kingdom | 3 |  |
| Lower and Middle Old Red Sandstone | Include sandstones, shale, mudstones, siltstones and limestones. | United Kingdom | 1 |  |
| Carboniferous: Dinantian and Namurian | Include sandstone, siltstone and mudstone. | United Kingdom | 1 |  |
| Upper Old Red Sandstone | Include sandstones, shale, mudstones, siltstones and limestones. | United Kingdom | 1 |  |
| Carboniferous: Westphalian | Include sandstone, siltstone and mudstone. | United Kingdom | 1 |  |
| Permian | Include sandstones, limestones, dolomites, and evaporites | United Kingdom | 3.5 |  |
| Upper Old Red Sandstone at Fife | Include sandstones, shale, mudstones, siltstones and limestones. | United Kingdom | 1 |  |
| Triassic and Permian | Include breccias and sandstones. | United Kingdom | 3.5 |  |

**Supplementary Table 1.** The collected mean USZ $v_{N}$ (m/year) in the USZ lithologies of different regions.
