# Supplementary material for: Nitrate transport velocity data in the global unsaturated zones
Source: Sci Data. 2022 Oct 11;9:613. doi: 10.1038/s41597-022-01621-x (PMC9553929; doi:10.1038/s41597-022-01621-x)
Supplement: Supplementary file 2 — Supplementary Table 2 [file 41597_2022_1621_MOESM2_ESM.docx]

| **GLiM lithology** | **United Kingdom** | **China** | **United States** | **Israel** | **Japan** | **Western Europe** |
| --- | --- | --- | --- | --- | --- | --- |
| Basic volcanic rocks | Permian(3.5) |  |  |  |  |  |
| Metamorphics |  |  |  |  | Mesozoic metamorphic and igneous rocks(1.65) |  |
| Unconsolidated sediments | Carboniferous: Westphalian(1) | Loess(0.24) | Loess(0.75) | Loess(0.66) |  |  |
| Siliciclastic sedimentary rocks | Lower Cretaceous Sands(3); Triassic Sandstones(3.5); Corrallian(1); Millstone Grit series of Cumbria, Durham and Northumberland(1); New Red Sandstone of SW England, Permian Sands of NW England(1.06); Millstone Grit Series(1); Purbeck Beds and Portland Beds(1); Upper Coal Measures: Pennant Sandstone of South Wales(1); Wealden: Hastings Beds(3); Oligocene: Bovey Beds(0.3); Lower and Middle Old Red Sandstone(1); Upper Old Red Sandstone(1);Triassic and Permian(3.5) |  |  |  |  | Triassic sandstone(3.5) |
| Mixed sedimentary rocks | Upper Lias: Bridport, Midford, Yeovil and Cotteswold Sands(0.1);Pliocene: Corralline Crag(3); Quaternary Norwich and Red Crags(3) |  |  |  |  |  |
| Carbonate sedimentary rocks | Chalk(0.95); Carboniferous: Limestone and Basal conglomerate(1); Cornbrash, Great Oolite and Inferior Oolite(1); Upper Lias: Bridport, Midford, Yeovil and Cotteswold Sands(0.1); Lower Carboniferous: Scremerston Group and Fell Saandstone of N England(1); Upper Greensand(3); Magnesian Limestone(10); Inferior Oolite: Lincolnshire Limestone(1.11); Cornbrash and Great Oolite of Lincolnshire(1.11); Whin Sill(1); Cornbrash, Great Oolite, Fullers' Earth and Inferior Oolite of S England(1.11); Carboniferous: Dinantian and Namurian(1); Permian(3.5); Upper Old Red Sandstone at Fife(1) |  |  |  |  | Chalk(1) |

**Supplementary Table 2.** The relationship between regional lithologies and GLiM lithologies. The number in parentheses is the mean velocity of each lithology.
