# Supplementary material for: Nitrate transport velocity data in the global unsaturated zones
Source: Sci Data. 2022 Oct 11;9:613. doi: 10.1038/s41597-022-01621-x (PMC9553929; doi:10.1038/s41597-022-01621-x)
Supplement: Supplementary file 3 — Supplementary Table 3 [file 41597_2022_1621_MOESM3_ESM.docx]

| **Zone** | **Region** | **Lithologies in Supplementary Table 1 and the GLiM lithologies** | $\boldsymbol{v}_{\boldsymbol{N}\boldsymbol{\_}\boldsymbol{base}}$  **(m/year)** | **The best mean** $\boldsymbol{v}_{\boldsymbol{N\_sim}}$  **(m/year)** | **The best** $\boldsymbol{R}$**(range)** | **Bias** |
| --- | --- | --- | --- | --- | --- | --- |
| 1 | China | Unconsolidated sediments | 0.24 | 0.240002 | 4.07562(2-6) | 0.000002 |
| 2 | USA | Unconsolidated sediments | 0.75 | 0.750013 | 0.824318(0.01-3) | 0.000013 |
| 3 | Israel | Unconsolidated sediments | 0.66 | 0.659958 | 0.54856(0.01-3) | 0.000042 |
| 4 | Japan | Metamorphics | 1.65 | 1.64999 | 36.9642(35-40) | 0.00001 |
| 5 | Europe | Triassic sandstone | 3.5 | 3.49972 | 0.767013(0.01-3) | 0.00028 |
| 6 | Europe | Chalk | 1 | 1.00003 | 2.8891(1-5) | 0.00003 |
| 7 | UK | Upper Lias: Bridport, Midford, Yeovil and Cotteswold Sands; | 0.1 | 0.1 | 25.7703(25-30) | 0 |
| 8 | UK | Oligocene: Bovey Beds | 0.3 | 0.299998 | 9.63424(7-13) | 0.000002 |
| 9 | UK | White Chalk Subgroup | 0.76 | 0.760001 | 21.9822(20-25) | 0.000001 |
| 10 | UK | Chalk | 0.95 | 0.950003 | 3.40684(2-5) | 0.000003 |
| 11 | UK | Carboniferous Limestone and Basal conglomerate; Cornbrash, Great Oolite and Inferior Oolite; Purbeck Beds and Portland Beds; Lower Carboniferous: Scremerston Group and Fell Saandstone of N England; Lower Carboniferous: Scremerston Group and Fell Saandstone of N England; Corrallian; Millstone Grit series of Cumbria, Durham and Northumberland; Millstone Grit Series; Purbeck Beds and Portland Beds; Upper Coal Measures: Pennant Sandstone of South Wales; Lower and Middle Old Red Sandstone; Carboniferous: Dinantian and Namurian; Upper Old Red Sandstone; Carboniferous: Westphalian; Upper Old Red Sandstone at Fife; | 1 | 0.999995 | 27.2585(25-30) | 0.000005 |
| 12 | UK | New Red Sandstone of SW England, Permian Sands of NW England; | 1.06 | 1.05999 | 3.41414(1-5) | 0.00001 |
| 13 | UK | Inferior Oolite: Lincolnshire Limestone; Cornbrash and Great Oolite of Lincolnshire; Whin Sill; Cornbrash, Great Oolite, Fullers' Earth and Inferior Oolite of S England | 1.11 | 1.11 | 2.98273(1-5) | 0 |
| 14 | UK | Lower Cretaceous Sands; Upper Greensand; Pliocene: Corralline Crag; Quaternary Norwich and Red Crags; Wealden:Hastings Beds; Quaternary Norwich and Red Crags | 3 | 2.99987 | 0.750223(0.01-3) | 0.00013 |
| 15 | UK | Triassic Sandstones; Permian; Triassic and Permian | 3.5 | 3.49997 | 0.439607(0.01-3) | 0.00003 |
| 16 | UK | Magnesian Limestone | 10 | 10.0004 | 0.960737(0.01-3) | 0.0004 |
| 17 | world | Basic volcanic rocks | 3.5 | 3.49989 | 0.713814(0.01-3) | 0.00011 |
| 18 | world | Metamorphics | 1.65 | 1.64999 | 12.6077(10-15) | 0.00001 |
| 19 | world | Unconsolidated sediments | 0.663 | 0.662969 | 1.6035(0.01-3) | 0.000031 |
| 20 | world | Siliciclastic sedimentary rocks | 2.57 | 2.56955 | 0.458313(0.01-3) | 0.00045 |
| 21 | world | Mixed sedimentary rocks | 2.03 | 2.02989 | 0.460777(0.01-3) | 0.00011 |
| 22 | world | Carbonate sedimentary rocks | 1.46 | 1.46001 | 1.48826(0.01-3) | 0.00001 |

**Supplementary Table 3.** The lithology, the best retardation factor, the best mean $v_{N\_sim}$, the $v_{N\_base}$, and their bias values in the 22 modelling zones.
